# Aichi Virus Induces Antiviral Host Defense in Primary Murine Intestinal Epithelial Cells

**DOI:** 10.3390/v11080763

**Published:** 2019-08-19

**Authors:** Yun-Te Chang, Ming-Hsiang Kung, Thung-Hsien Hsu, Wan-Ting Hung, Yao-Shen Chen, Li-Chen Yen, Tsung-Hsien Chang

**Affiliations:** 1 Department of Emergency Medicine, Kaohsiung Veterans General Hospital, Kaohsiung 81362, Taiwan; 2 Department of Physical Therapy, Shu-Zen Junior College of Medicine and Management, Kaohsiung 81362, Taiwan; 3 Department of Medical Education and Research, Kaohsiung Veterans General Hospital, Kaohsiung 81362, Taiwan; 4 Department of Critical Care Center Medicine, Kaohsiung Veterans General Hospital, Kaohsiung 81362, Taiwan; 5 Department of Internal Medicine, Kaohsiung Veterans General Hospital, Kaohsiung 81362, Taiwan; 6 Department of Internal Medicine, National Yang-Ming University, Taipei 12221, Taiwan; 7 Department and Graduate Institute of Microbiology and Immunology, National Defense Medical Center, Taipei 11490, Taiwan

**Keywords:** Aichi virus, intestinal epithelial cells, interferon, IRF3

## Abstract

The picornavirus Aichi virus (AiV) is a non-enveloped RNA virus that causes acute gastroenteritis symptoms, such as diarrhea, abdominal pain, nausea, vomiting, and fever. Antiviral host defense involves the fast response of type I interferon (IFN) and the secretion of inflammatory cytokines against pathogens. However, the intestinal inflammatory and antiviral response to AiV infection is poorly understood. This study evaluated the antiviral activity of intestinal epithelial cells (IECs), which form a single-cell layer separating the bowel wall from pathogens. Isolated primary mouse IECs were subjected to AiV infection and virion production, inducing the mRNA expression of type I/type III IFNs and inflammatory cytokines. The mechanism involved induced the expression of phospho-IFN regulatory factor 3 and mitochondrial antiviral-signaling protein of type I IFN signaling. These findings were also observed in AiV-infected human colon carcinoma cells. In summary, a viral productive and pathogenic infection of AiV in primary murine IECs is validated.

## 1. Introduction

Gastroenteritis refers to inflammation of the stomach comprising both small and large intestines. Viral gastroenteritis is caused by several viruses, such as rotaviruses, noroviruses, adenoviruses types 40 and 41, sapovirus, astroviruses, parechovirus, cosavirus, saffold virus and salivirus, and Aichi virus (AiV) [1,2]. AiV is a non-enveloped, small round virus of about 30 nm in diameter, belonging to the Kobuvirus genus of the *Picornaviridae* family. AiV causes gastroenteritis symptoms in humans, such as diarrhea, abdominal pain, nausea, vomiting, and fever [3,4]; however, AiV rarely causes lower respiratory tract disease [5,6]. Other kobuviruses members including sheep, bovine, porcine, murine, and canine kobuvirus have been identified [7,8]. Phylogenetic analyses revealed multiple cross-species transmission of kobuviruses both within and among mammalian species, which may cause sustained threat to public health [9].

AiV was first isolated in 1989 from the clinical specimen of a patient with oyster-associated gastroenteritis in Japan [6,10]. The virus is globally distributed and has been found in various environmental samples such as river water, groundwater, sewage, and shellfish [11,12]. Viral nucleic acid detection by PCR is a common method of AiV diagnosis [2]. A conventional method combining virus culture and immunofluorescence assay is an alternative for AiV diagnosis in the clinical laboratory [13].

Innate immunity involves the fast response of type I interferon (IFN) and secretion of inflammatory cytokines that can be triggered by the nucleic acid of virus replication products as well as Toll-like receptor (TLR) ligands, synthetic double-stranded RNA (poly(I:C), CpG dinucleotides, and lipopolysaccharide (LPS). Over the past decade, the interaction between viruses and host-type I IFN activity has been intensively studied to understand the host response to virus invasion [14,15,16,17]. 

Interferon regulatory factor 7 (IRF7) is a crucial regulator of type I IFN against pathogenic infection, which activates IRF7 by triggering signaling cascades from pathogen recognition receptors (PRRs) that recognize pathogenic nucleic acids [18]. The tripartite motif-containing protein (TRIM) family plays an important role in innate immunity [19]. TRIM21 is induced and interacts with IRF3 upon RNA virus infection. It positively regulates the strength and duration of primary antiviral response [20]; mouse Trim12c/Trim5 was also found to regulate type I IFN activity [21]. Moreover, retinoic acid-inducible gene I (RIG-I), melanoma differentiation-associated gene 5 (MDA5), Cxcl10, Viperin, and Mx1 are critical factors for a protective IFN response [22,23,24,25]. During virus infection, host cells immediately express IFN to restrict virus replication; however, many pathogenic viruses have devised evasion mechanisms to antagonize IFN production or activity, thus facilitating infection [26,27,28]. 

Intestinal epithelial cells (IECs) form a single-cell layer covering the intestine to absorb nutrients in the intestinal lumen. They also represent a barrier separating the bowel wall from fatal luminal pathogens that plays a critical role in the immune defense of our body [29]. TLRs are essential in pathogen recognition and bacterial clearance of leukocytes, but their dysregulation and unique signaling effects in IECs can have devastating consequences in an inflammatory environment. For example, TLR4 activation in IECs inhibits migration and proliferation of IECs but induces apoptosis of IECs, which promotes intestinal injury and inhibits intestinal repair [30,31,32]. 

An animal study revealed that activation of the TLR3-TRIF-caspase 8 signaling pathway by poly(I:C) in IECs substantially affected the structure and function of the small intestinal mucosa. Signaling through this pathway may have a host-protective role during infection with viral pathogens [33]. This hypothesis is partly supported in vitro, in that enterovirus 71 induced type I IFN and the expression of inflammatory cytokines as well as apoptosis in human colorectal adenocarcinoma HT29 cells [34]. Furthermore, rotavirus infection regulated type III IFN activity in IECs [35,36]. However, the interaction between AiV and intestinal innate immunity remains unknown.

This study investigated the activation of AiV-induced innate immune signaling and the inflammatory response in mouse IECs and human colon carcinoma cells for a better understanding of the pathogenesis and host response to this virus. 

## 2. Materials and Methods

### 2.1. Virus, Cell Lines, and Reagents

Human AiV was isolated from a newborn with diarrhea in Taiwan and the full genome sequence of the AiV has been deposited in GenBank (accession no. JX564249) [37]. AiV was propagated in Vero cells (ATCC: CCL-81), from which viral supernatant was harvested after removal cell debris by centrifugation. Human colon carcinoma cells, T84 (BCRC: 60149, Hsinchu, Taiwan) were grown in DMEM/F12 supplemented with 5% fetal bovine serum (FBS). The J774A.1 mouse macrophage cell line (BCRC: 60140) and African green monkey kidney epithelial cell line Vero (ATCC: CCL-81) were cultured in DMEM medium supplemented with 10% FBS (ThermoFisher Scientific, Waltham, MA, USA). TLR ligands, LPS of *Escherichia coli* (O111:B4, LPS-B4) and *Salmonella enterica* serovar Minnesota (LPS-SM) and poly(I:C) were from Sigma Aldrich (St. Louis, MO, USA).

### 2.2. Isolation of Mouse Intestinal Epithelial Cells

The animal experiments were conducted following the Animal Research: Reporting of In Vivo Experiments (ARRIVE) guidelines and approved by the institutional animal care and use committee of Kaohsiung Veterans General Hospital (VGHKS-2015-A025, VGHKS-2017-2020-A048). The purification of IECs was as described in [38] with modification. In brief, 6- to 12-week-old C57/B6 mouse intestines were opened longitudinally, washed in phosphate-buffered saline (PBS) and cut into 5-mm fragments. The epithelial integrity was disrupted by treatment with 1 mM dithiothreitol (DTT) on a shaker. Liberated IECs were collected and separated by Percoll gradient (Sigma Aldrich). Interface cells were collected and used as IECs. Purified IECs were cultured in high-glucose-formulated DMEM, supplemented with 10% FBS, 4 mM glutamine, 20 mM Hepes, 1 mM sodium pyruvate, and 100 U/mL penicillin/streptomycin. The culture medium and supplements were from ThermoFisher Scientific. Purified IECs were characterized using FACS analysis with antibodies against IEC markers, intestinal fatty-acid binding protein (I-FABP), and cytokeratin-18 (Cell Signaling, Danvers, MA, USA). Isolated IECs purity and survival rate were both >95%.

### 2.3. Aichi Virus Infection and Plaque Assay

Before virus infection, cells were replaced with serum-free medium, followed by addition of AiV at various multiplicities of infection (MOI). After 2 h adsorption (ADS) at 37 °C, the cell medium, after being washed, was replaced by culture medium. The infected cells were then incubated. To determine virus titers, culture media from AiV-infected cells was harvested for plaque-forming assays. Various virus dilutions were added to 80% confluent Vero cells and incubated at 37 °C for 2 h. After adsorption, cells were washed and overlaid with 1% agarose containing Eagle’s Minimum Essential Medium with 2% FBS. After a seven-day incubation, cells were fixed with 10% formaldehyde and stained with 0.5% crystal violet.

### 2.4. Immunofluorescence Assay

AiV infection was validated using immunofluorescence assay. Mock- or AiV-infected IECs were fixed with 4% paraformaldehyde for 30 min and then permeabilized with 0.5% Triton X-100 for 10 min, washed with PBS, and blocked with 10% skim milk in PBS for 30 min. The AiV capsid protein VP1 was detected with an anti-AiV VP1 antibody (1:500), which was described in our previous study [13], followed by Alexa 568-conjugated anti-rabbit IgG antibody (1:1000; ThermoFisher Scientific) each for 1 h in 25 °C. Cell nuclei were stained with 300 nM DAPI for 10 min. Fluorescence was observed under fluorescence microscopy (Zeiss, Axio Observer A1, Jena, Germany). 

### 2.5. Quantitative *Reverse Transcription-Polymerase Chain Reaction*(qRT-PCR)

The cDNA from LPS, poly(I:C)-stimulated IECs or J774A.1, mock or virus-infected IECs or T84 cells was synthesized from 0.5 μg total RNA using the SuperScript III reverse transcriptase kit (ThermoFisher Scientific). PCR amplification involved 3 ng cDNA in 10 μL fast SYBR Green PCR Master Mix (ThermoFisher Scientific) with 3 μM oligo primers in the ABI prism StepOne Plus Real-Time PCR System (ThermoFisher Scientific). The mRNA expression of host antiviral inflammation factors, such as, *Irf7, Ifnα, Ifnβ, Ifnλ2/3, Mx1, Viperin, Cxcl10, Rig-I, Mda5, Trim12c, Trim21, interleukin 6 (Il6), Il18* and *Tnfα* genes were measured. Transcript levels were normalized to that of glyceraldehyde-3-phosphate dehydrogenase (*Gapdh)* gene, a commonly used housekeeping gene. The relative abundance of mRNA transcripts was analyzed and calculated using the 2^ΔΔCT^ (where CT is the threshold cycle) method. Fold induction was compared with the control or mock group. 

To validate AiV genome replication in AiV-inoculated IECs and T84 cells, specific qRT-PCR primers for the AiV viral protein VP1-coding region (positive- and negative-strand RNA) or 3C-coding region were used [13]. Absolute quantification of AiV genome copy number was quantified using four series dilutions (100-fold each) of cloned AiV VP1 or 3C amplicons. All qRT-PCR primers were designed using software primer 3.0 (Applied Biosystems, Foster City, CA, USA), and the primer sequences are shown in Table S1.

### 2.6. Western Blot Analysis

IECs were lysed in RIPA buffer (150 mM NaCl, 0.5% sodium deoxycholate, 1% NP40, 0.1% SDS, 50 mM Tris-HCl [pH 8.0]) containing protease inhibitor and phosphatase inhibitor cocktail (Roche). Cell extract of 80 μg was separated by 10% SDS-PAGE and transferred to PVDF membranes, which were incubated with primary antibodies, then horseradish peroxidase-conjugated goat anti-rabbit IgG (Jackson ImmunoResearch Laboratory, West Grove, PA, USA) and visualized using an enhanced chemiluminescence system (ThermoFisher Scientific). Images were acquired using a digital image system (UVP BioSpectrum). The antibodies were anti-AiV VP1 [13], anti-phospho-IFN regulatory factor 3 (anti-phospho-IRF3, Abcam), anti-IRF3 (Santa Cruz Biotechnology, Houston, TX), anti-mitochondrial antiviral-signaling protein (anti-MAVS; Abcam, Cambridge, UK), and anti-β-actin (ThermoFisher Scientific) as the internal loading control.

### 2.7. Cell Viability and Cytotoxicity Assay

Proliferation of IECs was monitored using the WST-1 assay. Cells grown in 96-well plates were incubated with 10 μl WST-1 reagent (Roche, Basel, Switzerland) for 2 h. The absorbance at 450 nm was monitored and the reference wavelength was set to 620 nm. The cytotoxicity of AiV infection in IECs was measured using lactate dehydrogenase (LDH) cytotoxicity assay according to the manufacturer’s guidelines (LDH-Cytotoxicity Assay Kit II, Abcam). In brief, the culture medium from mock- or AiV-infected IECs or High Control, with cell lysis buffer was added to uninfected IECs and transferred into the 96-well plate (10 μL/well, triplicate). Then, 100-μL LDH Reaction Mix was added to each well, and the plates were incubated at room temperature for 30 min. Release of LDH was measured by absorbance at 450 nm and the reference wavelength was set at 650 nm. Low control was growth media. The percentage of cytotoxicity was calculated using the formula = (Test Sample − Low Control)/ (High Control − Low Control) × 100.

### 2.8. Statistical Analysis

Data of cell viability, cytotoxicity, qRT-PCR, In-Cell Western and plaque assay from three independent experiments were presented as mean ± SD. Student’s *t*-test was employed to determine the significance between treatment groups. Statistical significances were set as *, *p* < 0.05; **, *p* < 0.01; ***, *p* < 0.001.

## 3. Results

### 3.1. Isolation of Primary Mouse Intestinal Epithelial Cells for Toll-Like receptor-Ligand Stimulation

**Figure 1:**
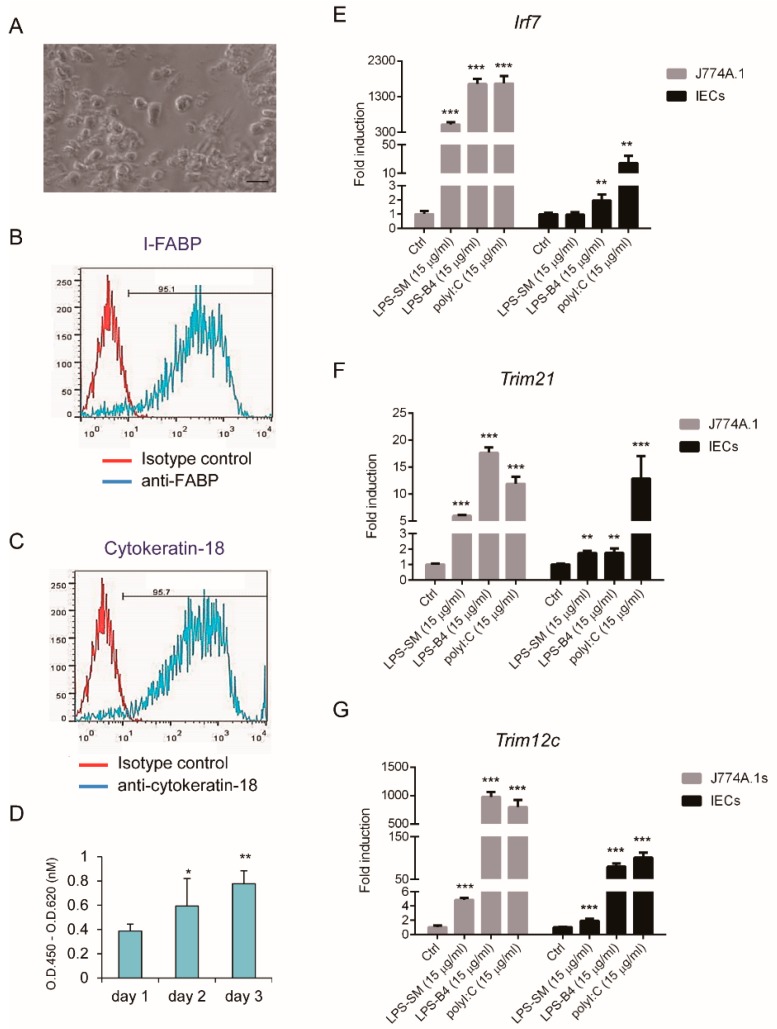
Isolation and cultivation of primary mouse intestinal epithelial cells (IECs). (**A**) Phase control image of IECs captured by microscopy, scale bar: 20 μm. (**B**,**C**) Expression of epithelial markers I-FABP and cytokeratin-18 on IECs cells analyzed using FACS analysis with anti-IFABP and cytokeratin-18 antibodies. (**D**) IECs (5 × 10^3^) grown in 96-well plate and their proliferation in day 1 to 3 cultures analyzed using WST-1 assay. (**E**–**G**) qRT-PCR analysis of mRNA expression of mouse *Irf7, Trim21*, and *Trim12c* in 1 × 10^6^ J774A.1 macrophages and IECs, stimulated with LPS of *Escherichia coli*. O111:B4 (LPS-B4, 15 μg/mL), *Salmonella enterica* serovar Minnesota (LPS-SM, 15 μg/mL) or poly(I:C) (15 μg/mL) for 24 h. Relative mRNA expression normalized to that of *Gapdh*, and the fold induction to untreated control. Data showed as means ± SD (*n* = 3). Student’s *t*-test *, *p* < 0.05; **, *p* < 0.01; ***, *p* < 0.001; compared with day 1 (**D**) or control (**E**–**G**), respectively.

To understand the antiviral activity of IECs, C57/B6 mouse IECs were isolated and cultured for TLR ligands and virus stimulation. Primary IECs showed adhesion morphology, as seen in Figure 1A. The epithelial features of IECs, the expression of I-FABP and cytokeratin-18, characterized by FACS analysis, showed that over 95% of cells were IECs, as seen in Figure 1B,C. The proliferation pattern of IECs was determined using the WST-1 assay, as seen in Figure 1D.

To understand the innate immune response, LPS (TLR2/4 ligand) and dsRNA (poly(I:C), TLR3 ligand) were used as stimuli. IRF7 is a crucial regulator of type I IFN against pathogenic infection and the TRIM family plays important roles in innate immunity; they are also inducible by LPS and poly(I:C) stimulation [21]. An examination of their mRNA expression showed expressions of Irf7, Trim21, and Trim12c in mice was significantly induced by LPS (TLR2/4 ligand) and dsRNA (poly(I:C), TLR3 ligand) in IECs, and control mouse macrophages, J774A.1 cells, as seen in Figure 1E–G. 

**Figure 2:**
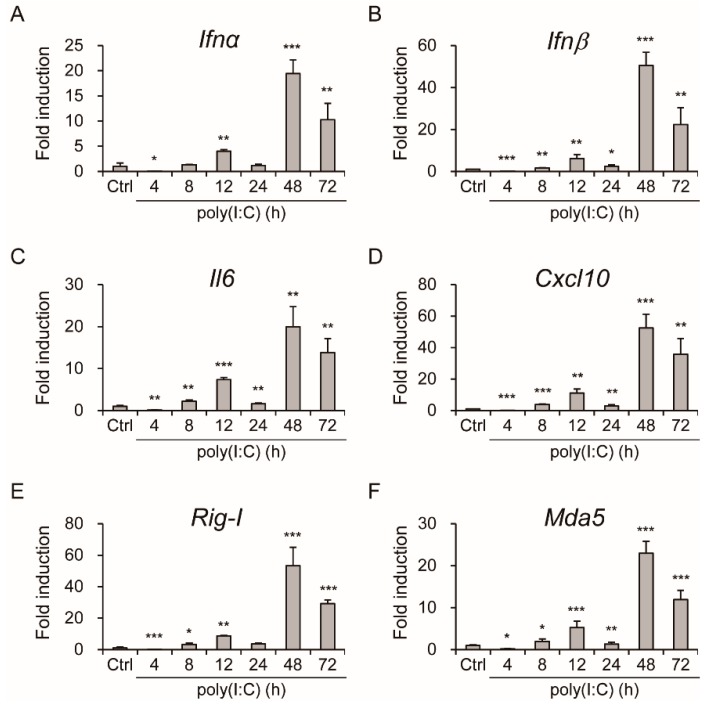
Expression of innate immune and inflammatory genes in poly(I:C)-stimulated IECs. (**A**–**F**) qPCR of mRNA expression of *Ifnα, Ifnβ, Il6, Cxcl10, Rig-I* and, *Mda5* at indicated times in IECs (1 × 10^6^) stimulated with poly(I:C) (15 μg/mL). Gene relative expression normalized to that of *Gapdh*. Data of fold induction compared with control showed as means ± SD (*n* = 3). Student’s *t*-test *, *p* < 0.05; **, *p* < 0.01; ***, *p* < 0.001; compared with control.

Two essential innate immune receptors, RIG-I and MDA5, detect viral double-stranded RNA in the cytoplasm. The inflammatory response triggered by these RIG-I-like receptors (RLRs) is one of the first and most important anti-infective lines of defense [22,39]. Analyzing the poly(I:C)-induced mRNA expression of *Ifnα, Ifnβ, interleukin 6 (Il-6), Cxcl10, Rig-I* and *Mda5* over time, as seen in Figure 2A–F, revealed activation of innate immunity by TLR ligands in IECs.

### 3.2. Productive Infection of Aichi Virus in Intestinal Epithelial Cells

**Figure 3:**
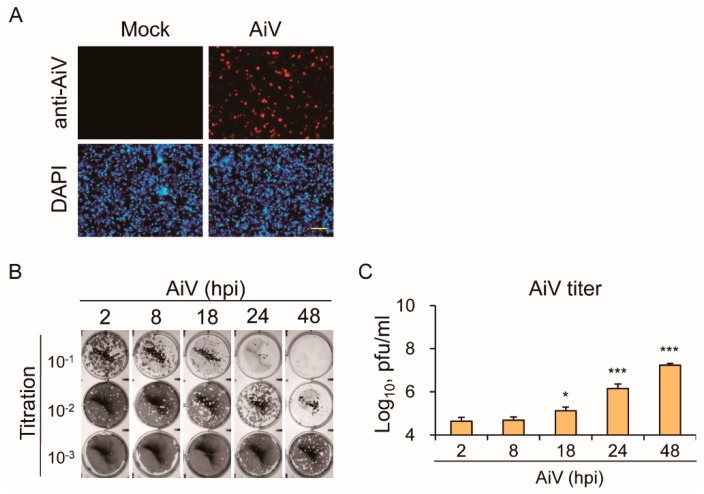
IECs are susceptible to Aichi virus (AiV) infection. (**A**) Immunofluorescence assay of IECs (1 × 10^6^) with mock infection or AiV infection at MOI of 5 for 24 hpi. AiV-infected IECs detected using the rabbit IgG-anti-AiV VP-1 antibody followed by goat anti-rabbit IgG Alexa 568. AiV-infected cells showed in red fluorescence. Cell nuclei showed in blue fluorescence of DAPI staining. Scale bar: 200 μm. (**B**) Culture medium AiV-infected IECs harvested for plaque assay, showing plague formation of AiV (10^−^^1^~10^−3^ titration). (**C**) AiV titers counted from plaque assay. Data of plaque titer compared with 2 hpi group showed as means ± SD (*n* = 3). Student’s *t*-test *, *p* < 0.05; ***, *p* < 0.001.

To test whether IECs are susceptible to AiV infection, IECs were infected with AiV at the multiplicity of infection (MOI) 5 for 24 h post-infection (hpi). When analyzed using immunofluorescence analysis with an anti-AiV VP1 antibody, AiV infectivity in IECs was about 22%, as seen in Figure 3A. To understand whether AiV established a complete life cycle for virus production in IECs, the culture medium from AiV-infected IECs was harvested for plaque assay. Plaque formation was found to increase over time, as seen in Figure 3B,C. Taken together, the expressions of viral protein and accumulated virions demonstrated an AiV-productive infection in IECs. 

### 3.3. Aichi Virus-Induced Type I Interferons and Expression of Inflammatory Cytokines in Intestinal Epithelial Cells

**Figure 4:**
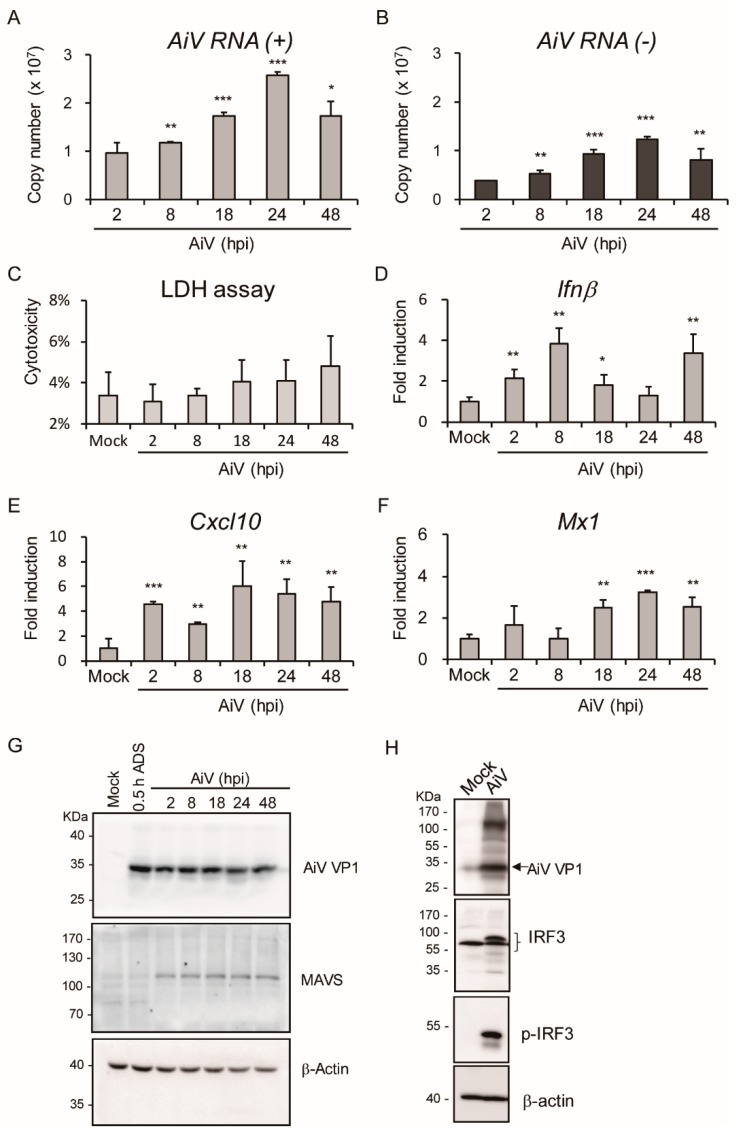
Kinetic analysis of AiV replication, cytotoxicity, and innate immune responses in IECs with AiV infection. (**A**,**B**) Absolute quantitation of dynamic viral genome copy number of AiV RNA (+) and (-) using RT-PCR with AiV VP1 (+) and (-) specific primers, respectively, in IECs (1 × 10^6^) with AiV infection at MOI of 5 for 2–48 hpi. (**C**–**G**) IECs were infected as described above, and (**C**) LDH release measurement of AiV cytotoxicity of AiV performed and measured as cytotoxicity (%). (**D**–**F**) qPCR analysis of mRNA levels of *Ifnβ, Cxcl10, and Mx1* genes normalized to that of *Gapdh*. Fold induction was compared with mock. Data compared with 2 hpi AiV (**A**,**B**) or mock (**C**–**F**) showed as means ± SD (*n* = 3). Student’s *t*-test *, *p* < 0.05; **, *p* < 0.01; ***, *p* < 0.001. (**G**,**H**) Western blot analysis of protein expression of AiV VP1, MAVS, phospho-IRF3, IRF3, and loading control β-actin in IECs with mock infection or AiV MOI 5 infection for 24 hpi or the indicated time course. ADS: adsorption.

To monitor dynamic viral replication in IECs, the replicon copy number of AiV genomic RNA was quantified using absolute quantification RT-PCR with AiV VP1 specific primers (positive- and negative-strand), as seen in Figure 4A,B (MOI = 5). Data of AiV RNA (+) and (-) indicated that the copy number of AiV replicons were enhanced from 8 to 48 hpi and peaked at 24 hpi. The pattern of AiV RNA replication level was also consistent with the accumulation of viral particles, as seen in Figure 3B,C. Moreover, the RNA expression level of AiV RNA (-) was observed at 2 hpi, suggesting the occurrence of AiV viral genome replication at the early stage of AiV infection in IECs. Data from LDH release assay showed that cell cytotoxicity, though increasing with the prolonged viral infection time, did not reach significant difference in IECs at MOI 5 for 2–48 hpi (48 hpi: 4.8% ± 1.5%, *p* value = 0.09), as seen in Figure 4C. In addition, the mRNA expression of host antiviral innate immunity genes *Ifnβ, Cxcl10*, and *Mx1* were induced by AiV infection, as seen in Figure 4D–F. 

Signaling pathway of host antiviral activity was investigated in view of the ability of AiV-induced type I IFN and cytokine mRNA expression. In particular, the MAVS–IRF3 pathway plays a critical role in regulating type I IFN production [40]. Both AiV infection-induced MAVS expression, as seen in Figure 4G, and IRF3 phosphorylation, as seen in Figure 4H, indicated the activation of antiviral innate immune signaling in IECs.

**Figure 5:**
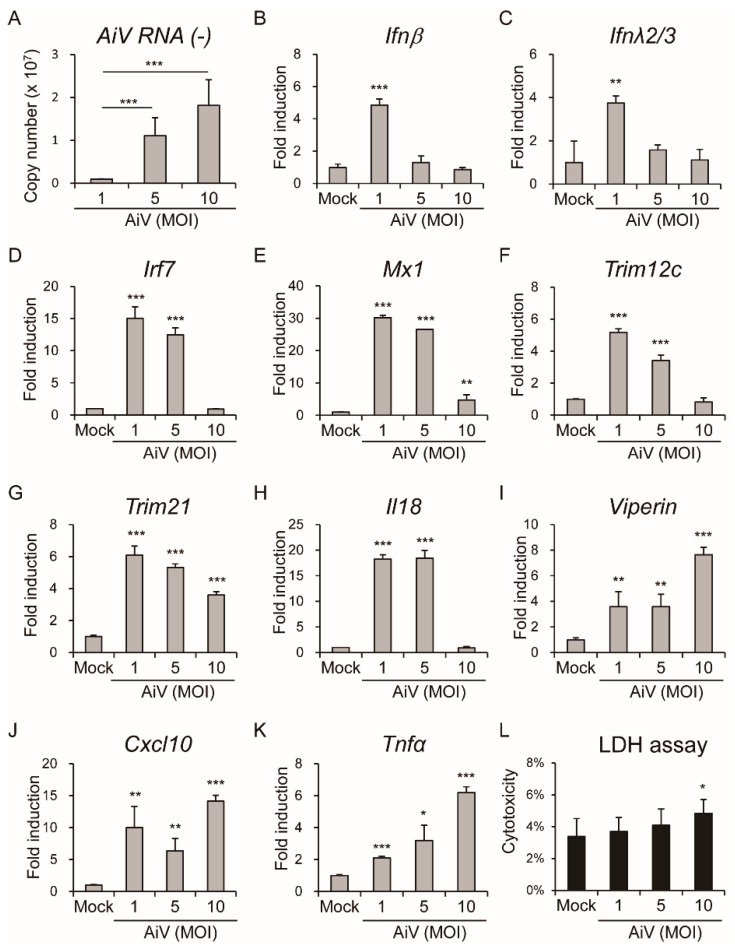
AiV infection induced innate immune response in IECs. (**A**) Absolute quantitation of the viral genome copy number of AiV RNA (-) using RT-PCR with AiV VP1 (-) specific primers, in IECs (1 × 10^6^) with AiV infection at the MOI of 1, 5, and 10 for 24 hpi. (**B**–**L**) IECs infected as described above, and (**B**–**K**) qPCR analysis of mRNA levels of *Ifnβ, Ifnλ2/3, Irf7, Mx1, Trim12c, Trim21, Il18, Viperin, Cxcl10,* and *Tnfα* genes normalized to that of *Gapdh*. Fold induction compared with mock. (**L**) LDH release measurement performed and measured as AiV cytotoxicity of (%). Data compared with uninfected mock showed as means ± SD (*n* = 3). Student’s *t*-test *, *p* < 0.05; **, *p* < 0.01; ***, *p* < 0.001.

To understand whether AiV infection induced the innate immune response in IECs, AiV replication as well as antiviral and inflammatory gene expression in IECs at different dosages of AiV infection (MOI = 1, 5, and 10) at 24 hpi were analyzed. Results are shown in Figure 5A and revealed a dose-dependent effect of AiV replication (AiV RNA negative-strand) in IECs. Moreover, mRNA levels of *Ifnβ, Ifnλ2/3, Irf7, Mx1, Trim12c, Trim21,* and *Il18* were highly induced in IECs with MOI = 1 or 5 infections but was decreased with MOI = 10, as seen in Figure 5B–H, implying modulation of host antiviral or inflammation responses by high titers of AiV. Nevertheless, the high MOI of AiV mediating a decreasing gene induction pattern was not seen in *Viperin, Cxcl10*, and *Tnfα*, which showed a high level of induction at MOI 10, as seen in Figure 5I–K. Thus, a MOI-dependent host response was indicated. To clarify whether high titer of AiV causes cell death, a cell cytotoxicity assay was performed. Results revealed lower cell cytotoxicity in high titer of AiV infection compared with the mock (MOI = 10: 4.8% ± 0.8% vs. mock: 3.4% ± 1.1%, *p*-value = 0.042), suggesting that AiV infection might cause modest cell death.

### 3.4. Aichi Virus Activated Antiviral Response in T84 Human Intestinal Epithelial Cells

**Figure 6:**
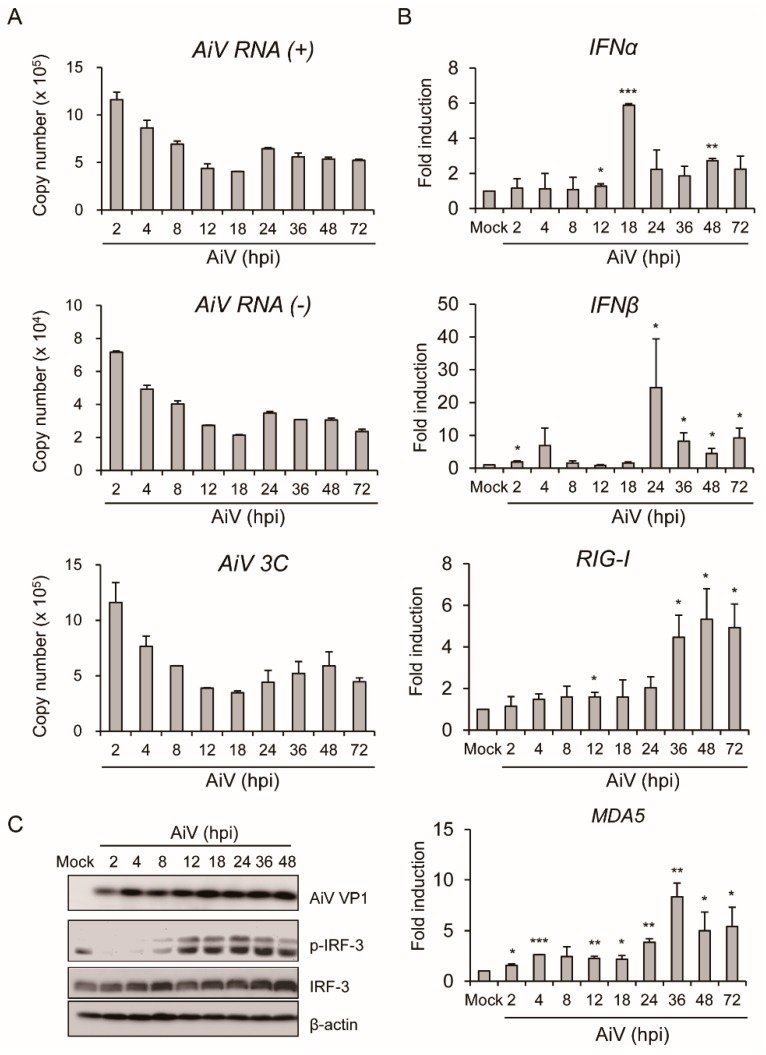
AiV activates type I IFN activity in T84 cells. (**A**) T84 (1 × 10^5^) cells infected with AiV at MOI 5. Quantitation of copy number of AiV viral gene (AiV VP1 (+), (-), and 3C-coding regions) replication and origin qPCR performed according to absolute quantitation at 2 to 72 hpi (*n* = 3, mean ± SD). (**B**) RT-PCR analysis of mRNA expression of *Ifnα, Ifnβ, Rig-I* and *Mda5* with AiV infection. Gene expression normalized to that of *Gapdh*. Data of fold induction compared with mock showed as mean ± SD (*n* = 3). Student’s *t*-test *, *p* < 0.05; **, *p* < 0.01; ***, *p* < 0.001; compared with uninfected mock. (**C**) Western blot analysis of the protein expression of AiV VP1, phospho-IRF3, IRF3 and loading control β-actin in IECs with mock infection and AiV infection (MOI = 5) for 2–48 hpi.

To verify activation of AiV-mediated innate immune in mouse IECs, T84 cells, which are a human intestinal epithelial cell line, were used as a human cell infection model of AiV. The previous studies in T84 cell indicated that the expression of antiviral/IFN response genes were up-regulated in response to poly(I:C) [41] and susceptible to AiV infection [13]. Dynamic replication of AiV genomic copy number was determined using qPCR with AiV VP1 (+), VP1 (-) and 3C-coding region specific primers at 2 to 72 hpi infection, as seen in Figure 6A. In addition, the antiviral response of *IFNα* and *IFNβ*, *RIG-I*, and *MDA5* mRNA expression were detected, as seen in Figure 6B. In contrast to a decrease in viral replication with infection time, type I IFN expression peaked at 18–24 hpi and decreased at the late phase of infection, 36–72 hpi. The contrasting pattern of viral gene and type I IFN expression might be attributed to the antiviral effect of type I IFN. Western blot analysis showed AiV-induced IRF3 phosphorylation, as seen in Figure 6C. These results are consistent with AiV-induced innate intestinal antiviral activity in mouse primary IECs. 

## 4. Discussion

AiV is a gastroenteritis virus. To understand its pathogenesis and fundamental viral immunity in the enteric system, the induction of intestinal innate immunity was evaluated using AiV in primary mouse IECs and human intestinal carcinoma cells. Both murine and human IECs were found to be susceptible to AiV infection. Moreover, antiviral and inflammatory activity in IECs was promoted by the infection. Mechanistic analysis revealed AiV-mediated MAVS expression and IRF3 phosphorylation, which verified the induction of host intestinal type I IFN-related antiviral effect. Along with the innate immune activation, modest cell death observed in AiV infection-mediated IECs evidenced an important defense mechanism for limiting AiV replication in the intestinal tract, which may alleviate intestinal damage and maintain normal physiological functions of the intestinal tract. This study provides useful in vitro data for additional investigations of intestinal innate immunity in the mouse infection model.

PRRs, sensing microorganisms in the intestine, induce sophisticated signaling in the intestinal mucosa, which is required to sustain the integrity of the intestinal barrier and immune homeostasis. The PRR signaling pathway also triggers an innate immune response against invasive pathogens in the gut [22,42]. The effect of poly(I:C) induction of innate immunity against AiV infection was revealed [13]; our finding on the response in IECs with LPS and poly(I:C) stimulation supports the hypothesis of an intestinal-mucosa innate immunity, as seen in Figure 1E–G and Figure 2. 

For decades, intestinal cancer cell lines have been broadly used for intestinal epithelial experiment due to their immortality and ease of cultivation. However, with the inherent biological abnormalities of cancer cell lines in comparison with normal cells, significant limitations are associated with the cancer cell lines [43,44]. To overcome such shortcomings, advanced techniques to culture primary IECs were developed [38,45,46,47]. Our culture of primary murine IECs was effective because they responded to TLR ligand stimulation and AiV infection, as seen in Figure 1, Figure 2 and Figure 3. In addition, the long-term culture of primary murine IECs is susceptible to rotavirus infection [48]; therefore, culture of murine primary IECs is useful for investigating virus–host interaction in vitro.

Type I IFN inhibits AiV and enterovirus 71 (EV71) [13,49], thus, AiV-induced type I IFN expression in IECs might be critical in the gut for enteric virus restriction, as seen in Figure 4, Figure 5 and Figure 6. This result was supported by the observation of EV71-induced antiviral response and cytopathic effects in human intestinal HT-29 ECs [34]. Activation of EV71-mediated inflammation in HT29 cells was detected in our AiV-infected primary IECs and T84 cells [34], as seen in Figure 4, Figure 5 and Figure 6. As seen in Figure 5, results showed revealed induction of antiviral-inflammatory signaling by AiV infection; in contrast, high titer of AiV infection (MOI = 10), which causes modest cell death, inhibits gene expression of the IFN pathway without affecting AiV-induced *Tnfα, Viperin*, and *Cxcl10* gene expression. However, poly(I:C)-induced IEC apoptosis is independent of TNF and type I IFN signaling [33]. Antiviral and inflammation activities in host could not inhibit initial AiV replication and would be harmful to IECs, despite the cell damage acting as another defense mechanism to restrict AiV invasion. Inflammation might play a key role in intestinal pathogenicity [50,51]. 

Recent studies on the control of viral infection by type I and III IFNs have revealed distinct roles for these cytokines in the gut at other barrier surfaces. Type I IFN is essential in the localized control of infection at most tissue barriers. Type III IFNs, such as IFN-λs, are important in the localized control of infection at the mucosal epithelium [17]. In the mouse model, IFN-λ has shown antiviral potential against enteric viruses such as norovirus and rotavirus [52,53]. Although *IFNλ2/3* expression was detected in AiV-infected mouse IECs, as seen in Figure 5C, the effect and mechanism of type III IFNs against AiV or other picornaviruses remain unknown. Thus, the illustration of the interaction between AiV and type III IFN in the gut merits further elucidation.

This study revealed the effect of AiV infection in primary mouse IECs and human colon cells. Findings obtained broaden our understanding of the host defense mechanism and pathogenesis of AiV infection.

## Supplementary Materials

The following are available online at https://www.mdpi.com/1999-4915/11/8/763/s1, Table S1: The list of PCR primer sequences.
