# Supplementary material for: Aichi Virus Induces Antiviral Host Defense in Primary Murine Intestinal Epithelial Cells
Source: Viruses. 2019 Aug 19;11(8):763. doi: 10.3390/v11080763 (PMC6722774; doi:10.3390/v11080763)
Supplement: Supplementary file 1 [file viruses-11-00763-s001.pdf]

Table S1. The list of PCR primer sequences

Target gene	Primer sequences	
murine Gapdh	F	5′-CATGGCCTTCCGTGTTCCTA-3′
	R	5′-GCGGCACGTCAGATCCA-3′
murine Ifna	F	5′-AGGCTCTGTGCTTTCCTGATGA-3′
	R	5′-TCACATCCTAGAGAGCAGGTTGAC-3′
murine Ifnb	F	5′-CCCTATGGAGATGACGGAGAAG-3′
	R	5′-GAG CAT CTC TTG GAT GGC AAA-3′
murine Ifnλ 2/3	F	5′-AGC TGC AGG TCC AAG AGC G-3′
	R	5′-GGT GGT CAG GGC TGA GTC ATT-3′
murine Irf7	F	5′-AGC TGT GCT GGC GAG AAG-3′
	R	5′-TGG AGT CCA GCA TGT GTG TG-3′
murine Mx1	F	5′-TGC TGT ACT GCT AAG TCC AAA-3′
	R	5′-GCAGTAGACAATCTGTTCCATCTG-3′
murine Trim12c	F	5′-GGC TGC ATC ACG TTG TAC TTT G-3′
	R	5′-CCACATGTAGATTAGGCCTCAGATT-3′
murine Trim21	F	5′-ATG TGG CCA TTG CTT TTG C-3′
	R	5′-GCC CCC ATT CTT CCC AAC T-3′
murine Il6	F	5′-CCACGGCCTTCCCTACTTC-3′
	R	5′-TTGGGAGTGGTATCCTCTGTGA-3′
murine Il18	F	5′-AAATGGAGACCTGGAATCAGACA-3′
	R	5′-TTCCGTATTACTGCGGTTGTACA-3′
murine Viperin	F	5′-TGC TAT CTC CTG CGA CAG CTT-3′
	R	5′-CCT TGA CCA CGG CCA ATC-3′
Murine Cxcl10	F	5′-CGA TGA CGG GCC AGT GA-3′
	R	5′-CGCAGGGATGATTTCAAGCT-3′
murine Tnfα	F	5′-CAGCCGATGGGTTGTACCTT-3′
	R	5′-GGC AGC CTT GTC CCT TGA-3′
murine Rig-I	F	5′-GGCATGGACTGTGGTCAT-3′
	R	5′-GTTTAACCCCTCCCCACCAT-3′
murine Mda5	F	5′-GCAACAAGTACTCAACCACTAAACCA-3′
	R	5′-AGGAGATGAACTTGACTGTGGAAAT-3′
human Gapdh	F	5′-TGCACCACCAACTGCTTAGC-3′
	R	5′-GGCATGGACTGTGGTCAT-3′
human Ifna	F	5′-CCT CGC CCT TTG CTT TAC TG-3′
	R	5′-CAG AGA GCA GCT TGA CTT GCA-3′
human Ifnb	F	5′-TGA GCA GTC TGC ACC TGA AA-3′
	R	5′-GCT TGA AGC AAT TGT CCC GT-3′
human Rig-I	F	5′-GCA GAG GCC GGC ATG AC-3′
	R	5′-TGTAGGTAGGGTCCAGGGTCTTC-3′
human Mda5	F	5′-TGCTGGACTACCTGACCTTTCTG-3′
	R	5′-GGCGACTGTCCTCTGAATCTG-3′
AiV VP1(+)	F	5′-AGA ATG GTG CAG CTG ACA ACT C-3′
	R	5′-GGG ATT TTC AGT GTA GTC GAA GGT-3′
AiV VP1(-)	F	5′-GAA GCG ATC CCG GAA ACA T-3′
	R	5′-AGG CAC AAT CAT CCC ACT CAA C-3′
AiV 3C	F	5′-CCC CAC TGG ACT CCT TGT TTC-3′
	R	5′-TGT CGG AGA TGC GGA CAT AG-3′

F: forward primer; R: reverse primer
